# Affordance trajectories and the usefulness of online records access among older adults in Sweden

**DOI:** 10.1177/20552076241287354

**Published:** 2024-10-21

**Authors:** Isto Huvila, Hanife Rexhepi, Jonas Moll, Maedeh Ghorbanian Zolbin, Charlotte Blease, Annika Bärkås, Rose-Mharie Åhlfeldt, Josefin Hagström, Bridget Kane, Isabella Scandurra, Maria Hägglund, Gunnar O. Klein, Bo Wang, Anna Kharko

**Affiliations:** 1 Department of ALM, 8097 Uppsala University, Uppsala, Sweden; 2 School of Informatics, University of Skövde, Skövde, Sweden; 3 Centre for Empirical Research on Information Systems, School of Business Örebro, University Örebro, Sweden; 4 Department of Computer Science, Aalto University, Espoo, Finland; 5 Participatory eHealth and Health Data Research Group, Department of Women's and Children's Health, Uppsala University, Uppsala, Sweden; 6 Medtech Science & Innovation Centre, Uppsala University Hospital, Uppsala, Sweden; 7 Digital Psychiatry, Department of Psychiatry, Beth Israel Deaconess Medical Center, Harvard Medical School, Boston, USA; 8 Business School, Karlstad University, Karlstad, Sweden; 9 Norwegian Centre for E-Health Research, University Hospital of North Norway, Tromsø, Norway; 10 School of Psychology, Faculty of Health, University of Plymouth, Plymouth, UK

**Keywords:** Patient-accessible electronic health record, online record access, older adults, national survey, human–computer interaction, usability

## Abstract

**Objective:**

The current understanding of the breadth of individual differences in how eHealth technologies are perceived as useful for different purposes is incomprehensive. The aim/purpose of the study is to improve the understanding of diverse perceptions of the usefulness of technologies by exploring older adults’ use of their patient-accessible electronic health records (PAEHRs).

**Methods:**

The study applies and extends Affordance Theory based on an empirical analysis of data from the NORDeHEALTH 2022 Patient Survey on attitudes toward PAEHR in Norway, Sweden, Finland, and Estonia. Responses from 3964 participants in Sweden, aged 65 + years were analysed. Data included demographics and agreement ratings to reasons for using PAEHR. To analyse variation in the reasons for using PAEHR, group comparisons were conducted based on gender (male/female), age group (65–74, 75–84 and 85+) and earlier encouragement to use PAEHR.

**Results:**

Overall, the findings suggest that PAEHRs have multiple parallel affordance trajectories and affordance potencies that actualise differently depending on needs. The top reasons, pointing to both orientational and goal-oriented affordances for using PAEHR, were improving understanding of health issues, getting an overview of medical history/treatment and ensuring understanding of what the doctor said. Men reported more often sharing information with relatives or friends as a reason to access PAEHR. Women were more inclined, albeit similarly to men less frequently, to read their PAEHR for detecting errors. Age had little influence on reasons for using PAEHR.

**Conclusions:**

The study applies and extends Affordance Theory in the context of older adults’ PAEHR use based on findings from the largest national investigation of reasons for older users to access PAEHR in Sweden demonstrating the applicability of the theory in improving the understanding of the diversity of individual perceptions on eHealth technologies.

## Introduction

Older adults’ information and communication technology (ICT) adoption is increasing and perceived to have a positive impact on their everyday lives.^1^ eHealth services can improve access and provision of care,^2^ especially when online service use increases among older adults.^3,4^ Patient-accessible electronic health records (PAEHRs) have become a widespread eHealth tool for enabling patient access to information in their healthcare records. Evidence suggests that the benefits to patients outweigh the risks of patients getting confused by their records.^5^ Patients using PAEHR report that reading their notes engages them in their care, improves health management and increases their sense of control over their health.^6–8^

Recent PAEHR research has focused on patients’ and healthcare professionals’ viewpoints. However, to date, few studies provide insights into older adults’ PAEHR use. Older adults are known for their limited PAEHR use, despite anticipated benefits,^9,10^ owing to their age and the likelihood of suffering from chronic conditions. Various studies, for example, in Sweden^11,12^ and Norway,^8^ show that older adults are less likely to access their PAEHR, but those who do, benefit more.^13,14^ Whereas younger adults can be more interested in PAEHR, older individuals access it to understand their health condition, prepare for visits and get involved in their own healthcare. The problem statement underpinning this study is that the variation in how older adults use and perceive PAEHRs and the types of information they find useful is poorly understood considering the apparent risk of healthcare disparities among older adults. This question links to a broader issue of understanding the breadth of individual differences in how technologies are perceived as useful for various purposes.

The aim of this study is to improve our understanding of the diverse perceptions of the usefulness of PAEHR, this article reports affordances of online electronic healthcare records as perceived by Swedish adults, 65 years or older, that is, what offerings of PAEHRs they find useful. The study addresses two research questions: (RQ1) what are the reasons given by Swedish older adults’ for reading their online record information? And (RQ2) how can the variation in older adults’ responses be explained from the perspective of the perceived opportunities (commonly termed, “affordances”) provided by PAEHRs?

Empirical findings are interpreted from the perspective of Affordance Theory^15,16^ to interrogate how older adults of the three investigated age groups perceive what PAEHR offers them. Rather than assuming that the PAEHR offers the same opportunities for everyone, the novelty of this article is to advance and nuance the understanding of the PAEHR as a technological and informational artefact with widely diverging offerings to individuals in different ages and life situations and explore how conflicts, positive and negative experiences can be explained in terms of different, sometimes conflicting, perceived affordances (i.e. perceived opportunities offered by PAEHRs)^16^ and how they actualise (or not) in practice as affordance potencies (i.e. “the strength of the relationship between the abilities of the individual and the features of the system at the time of actualization, conditioned by the characteristics of the work environment”).^17^ Our study also advances the conceptual understanding of the potencies and trajectories of how specific affordances actualise for different individuals and how they can function as stepping stones for each other.

### Literature review

#### Older people and technology

Technology use has increased significantly among older adults during the last decade.^1^ User-centred design, attentiveness to older adults’ perspectives^18^ and improved familiarity of older adults with ICT have been major contributors.^19^ In addition, older adults’ capacity to manage their lives and meet their daily needs with technology use is higher than before.^20^ Older adults are better in progressing with their care at their own place and pace and accommodating their health issues independently with digital health services.^21^ Most older adults also prefer to maintain an independent lifestyle.^18^

Despite the major advances in technology development, older adults still use internet-enabled devices less compared with younger generations.^22^ Some are unenthusiastic about technology and consider it ‘non-essential’.^23^ Furthermore, insufficient technical skills and education limit immersion in new or unfamiliar technologies.^24–26^ The fast pace of technological development, often perceived as complex, can foster negative attitudes and reduce willingness to explore technology use.^27,28^ Some consider technology untrustworthy, especially with sensitive and important issues like health.^29^ Uncertainty about communication in emergencies or with mental health issues increased preference for face-to-face meetings with healthcare professionals.^2^ Older adults often suffer from physical and cognitive decline, which may compromise their ability to use technology.^25,30^

Combined, these challenges increase the likelihood of an age-related ‘digital divide’ between those with access to technology, knowledge to use it and the ability to engage with and benefit from it^31^ and those without,^23^ which may lead to social isolation and lack of social support among non-users. Berg-Weger and Morley^32^ suggest that this digital divide might be narrowed through proper interventions and encouragement from professional or personal networks. A narrative review of the literature addressing patient perspectives on digital health tools by Madanian et al.^26^ highlight the importance of patient acceptance and active involvement in the design and implementation of ICT to help increase patients’ engagement with emerging technologies. Moreover, adults are a highly divergent group with respect to technology access and digital health literacy, which affects their use of eHealth services.

#### PAEHRs

That PAEHRs are now increasing is reflected in the growing corpus of studies worldwide, including from the Nordic countries^8,12,33^ and England^34^ to the USA.^7,35^ In Sweden, patients can access their PAEHR, named *Journalen*, through the national patient portal 1177.se. Sweden has approximately 10 million inhabitants, and by February 2023, the total number of unique *Journalen* users reached six million. Through *Journalen*, patients obtain a variety of information on care, including clinicians’ notes, lists of prescribed medications, lab results, diagnoses, referrals and vaccinations.

Patients accessing their PAEHR report using it to become more involved in their care, to prepare for visits, follow-up on test results, get an overview of their healthcare visits, and so on.^12^ In a qualitative interview study by Rexhepi et al.,^36^ it was shown that access to test results was one of the main reasons why cancer patients wanted to access the PAEHR. Survey studies report additional benefits of accessing PAEHR, such as improved understanding of care plans,^7,37^ greater trust in healthcare provider^38^ and enhanced control in health management,^7,8^ including medications.^39,40^ Some studies found improved communication with clinicians.^41^ However, healthcare professionals are often critical of PAEHR. Physicians, especially in mental healthcare,^42^ expressed concerns that PAEHRs increase patient anxiety, incur risks to patient safety, increase professionals’ workload, reduce efficiencies and increase burnout.^43–45^

#### Older people and PAEHR

In the context of PAEHRs, few studies have examined the views and experiences of older adults reading their records online, however, with findings that point to differences and trajectories in how PAEHR use changes. In the USA in 2021, a secondary analysis of the largest patient survey conducted into users’ experiences with reading their online visit notes, known as Open Notes, found that older patients (aged 65 years and over) with more than two chronic conditions were more likely than those with fewer or no chronic conditions to report that access helped them remember their care plan, take their medications as prescribed, understand and feel more in control of prescribed medications; few respondents reported feeling worried or confused about their health or medications after access.^46^ A Finnish interview study with 24 respondents, Eriksson-Backa et al.^33^ reported the most commonly used features in the PAEHR *MyKanta* among older patients were e-prescriptions, test results and medical reports. Furthermore, *MyKanta* use had an impact on either the interviewees’ health behaviour or personal health information management. For some users, PAEHR use improved their understanding of their health, consistent with other surveys.^12,37,46,^^47^

eHealth service use expanded in healthcare during the COVID-19 pandemic.^48^ Rapid uptake of telehealth and, for example, the scheduling and provision of COVID-19 test results meant that patient portals played a prominent role throughout the pandemic.^49^ While age is generally a strong indicator of differences in digital service use, older adults are a highly divergent group with respect to technology access and digital health literacy, which affects their use of eHealth services.^25^

Some studies have identified factors that might hinder PAEHR use, including sociodemographic background,^50^ health literacy,^51,52^ and technology skills.^53^ Blease et al.^6^ hypothesised that some patient populations may experience more pitfalls in communication during face-to-face visits rendering them more likely to suffer health disparities. They proposed PAEHRs as a novel ‘work around’ helping to solve patient-specific challenges with traditional one-shot, time-dependent visits. For example, compared to the younger, the older patients may have more information to remember but poorer recall; PAEHRs may, therefore, function to elongate the visit, helping to supplement compromised recall about what was said.^6^

A part of the studies have also made explicit comparisons between age groups. Taha et al.^52^ explored the ability of middle aged (40–59 years) and older adults (60–85 years) to use a personal health record to perform common health management tasks. The respondents, regardless of age group, expressed interest in using their record and found it useful. However, they had difficulty navigating the system and made errors in performing health management tasks.^52^ Similar results were reported by Wildenbos et al.^54^ in the Netherlands where older adults’ patient portal registration rates were collected from the electronic health record (EHR) database. In a survey study with 1155 Swedish respondents Huvila et al.^47^ found that adults under 51 years old were more likely to use PAEHR for general interest rather than for satisfying specific information needs. They valued online access to information less than adults aged 51–66 years old who were more likely to report using the PAEHR to understand their health condition, prepare for visits and to become involved in their own healthcare. Older respondents (aged 66 years and over) were more likely to consider PAEHR information as useful but experienced the technology difficult to use. The authors suggest that the findings point to trends in how PAEHR preferences change from perceiving it as a source of general information to means of managing personal health.^47^

As a whole, the earlier research shows that technologies and eHealth in general, and PAEHR in particular have diverse offerings to older adults. The specific opportunities perceived vary between individuals and groups. There are indications that the opportunities eHealth technologies offer to their users might evolve when individuals get older but also that the variation depends on various demographic and practical factors.

### Theory of affordance

In order to investigate closer the differences and trajectories of the perceived offerings of eHealth technologies and more specifically the PAEHR, this study inquires into the potential affordances of PAEHR that individuals in the three older age groups find most relevant to them. The inquiry is guided by the theory of affordances, defined as ‘the possibilities for action’, pioneered by Gibson.^15^ The theory has gained popularity across fields from psychology, human–computer interaction, to health informatics. In information technology research, the affordance lens is typically used to understand the relationship between technology and human actors.^55^ In this context, the affordance concept is geared toward technologies. Markus and Silver^56^ defined affordance as ‘the possibilities for goal-oriented action afforded to specified user-groups by technical objects’. Majchrzak et al.^57^ advised that ‘by looking at technologies as sets of affordances and constraints for particular actors, IS [information systems] researchers can explain how and why the “same” technology is used or has different outcomes in different contexts’, suggesting that the notion of affordance can help understanding potential technology use and the barriers and difficulties in applying it in specific contexts.

Rather than being static over time and contexts, based on the earlier research we presume that the affordances of PAEHR change over time and depend on individuals’ abilities, features of PAEHR and the contexts and situations of its use. This is reflected in Anderson and Robey's^17^ concept of affordance potency (how well an object or feature communicates its use to a person. High affordance potency means that it is very obvious what actions can be taken with an object). Affordance potency is situational and can change over time as abilities, features and the environment evolve.^17^ Hence, affordance potency can help explain ‘why unique individuals encounter difficulties during actualisation and how [those encounters] influence their decisions to actualise an affordance or to find ways to work around the difficulties’.^17^

In an earlier article, Wang and Zhao^58^ approached PAEHR using the affordance lens and found that older adults’ attachment to the platform, and doctors, positively influences usage intention. They also report that the affordance of the aggregation and interactivity of PAEHRs is positively related to elders’ attachment to the platform.

In contrast to studying the effect of affordances on usage intention, we use and extend the theory to help understand the functions of the PAEHR and information that older adults find useful. The concept of affordance potency as an integral theoretical model of affordances is used to explain the relationship between PAEHR users and system features in terms of potentials and barriers of PAEHRs. Moreover, we strive to identify different types of affordances that the PAEHR offers for various individuals, including goal-oriented or functional affordances that support specific health and care-related goals, orientational affordances that satisfy general curiosity and provide orientation,^56^ cognitive affordances that help individuals to think and understand^59^ and mnemonic affordances that support individuals in remembering.^60^

This study advances the Affordance Theory in the context of technology and health research by interrogating how affordances of particular technologies differ for various individuals, how perceived affordances may be in conflict with each other and how affordance potencies sometimes are realised when, as Leonardi^61^ puts it, human and material agencies involved imbricate, that is, overlap in regular patterns that shape an infrastructure that produces, sustains or changes technologies or how people act.

## Methods and material

In the NORDeHEALTH project,^62^ the NORDeHEALTH 2022 Patient Survey was conducted to explore the opinions and experiences of PAEHR users in Estonia, Finland, Norway and Sweden. The present study utilises the responses collected in Sweden only. The data were collected using an online survey questionnaire for 3 weeks from 23 January to 13 February 2022, after research ethical approval was granted by the Regional Ethical Review Board, Uppsala, Sweden (EPN 2021/05229). Participants were informed of the study and gave their written informed consent to participate at the opening the survey by answering a question on that they had received the written information on the study provided in the beginning of the questionnaire, understood that they had a possibility to ask questions through contacting the research team and consented their participation and processing of the data they provided. No tools or questionnaires with copyright restrictions were used in the study. Target participants in the survey were individuals aged 15 years and above, who logged into *Journalen*, the PAEHR service in the web patient portal 1177.se available for everyone with a Swedish personal identification number, during the data collection period. As no preset quotas for respondents were set, convenience sampling was employed. To ensure that only verified users took part in the study, the survey advertisement link was placed in *Journalen* and only accessible upon authentication. Participation was anonymous, voluntary and not compensated. Only fully answered surveys were recorded. The survey design and distribution strategy are described in more detail elsewhere.^63^

**Figure 1.**
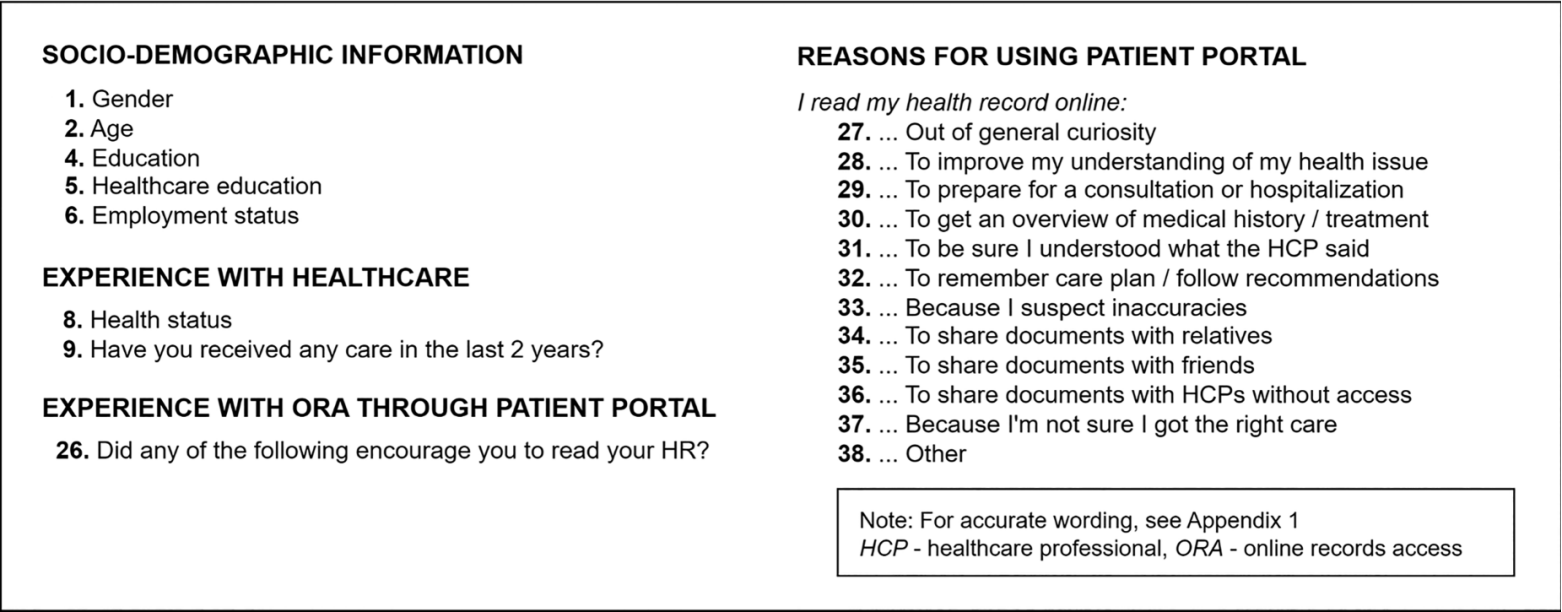
NORDeHEALTH 2022 Patient Survey items used in the analysis.

The NORDeHEALTH 2022 Patient survey consisted of 85 survey items distributed under seven thematic sections including both closed questions with given response options (set categories, yes/no or 5-point Likert scales) and free-text questions. This analysis focuses on 24 from four sections (see Figure 1).

The perceived affordances of the PAEHR and their strength were measured by asking the respondents to what degree they agreed (on 5-point Likert scale) that they read their PAEHR for 12 reasons specified in the questionnaire (Figure 1). The set of measured reasons was developed on the basis of the earlier literature^64–66^ and from the outset of the Affordance Theory interpreted as indications of respectively goal-oriented or functional affordances, orientational affordances, cognitive affordances and mnemonic affordances (Table 1). Questionnaire items used in this study do not comprise validated scales.

**Table 1.** Descriptive statistics of the reasons to read EHR online.

Type of Affordance	Affordances: I read my health record online …	Mean	Mode	Std. Deviation
Orientational affordance	…to improve my understanding about my health issue.	4.397	5	1.043
Orientational affordance	… to get an overview of my medical history and/or treatment.	4.277	5	1.079
Cognitive affordance	… to be sure I understood what the doctor said.	4.119	5	1.233
Goal-oriented/functional affordance	… to prepare myself for a consultation or hospitalization.	3.827	5	1.397
Mnemonic affordance	… to remember the care plan/follow my treatment recommendations.'	3.381	5	1.504
Orientational affordance	…out of general curiosity.	3.059	5	1.575
Goal-oriented/functional affordance	…to share documents with health professionals who do not have access.	2.137	1	1.445
Cognitive affordance	…because I am not sure if I got the right care.	1.933	1	1.263
Goal-oriented/functional affordance	…to share documents with relatives.	1.882	1	1.279
Cognitive affordance	…because I suspect inaccuracies.	1.879	1	1.246
Goal-oriented/functional affordance	…to share documents with friends.	1.414	1	0.894

EHR: Electronic health record.

Similarly, based on the literature, we included a set of dependent variables, selected for the study as potentially relevant to eliciting the variation of affordances of PAEHR including demographic information (gender, age, education-level, health education, employment and health status) and whether a healthcare professional had encouraged PAEHR use.

To indicate their age group, the respondents could check the appropriate age interval, by selecting from the “65–74 years”, “75–84 years” and “85 years and older”. In total, 3964 respondents (30.5% of 13,008 survey respondents) were 65 years or older. All of them were included in the study. The demographic distribution among the respondents selected for the study is summarised in Table 2.

**Table 2.** Respondent demographics (N = 3964).

Variable	Categories	N	%
Gender	Women	2031	51.2
	Men	1933	48.8
	Other	–	–
Age	65–74 years	2612	65.9
	75–84 years	1262	31.8
	85 years or older	90	2.3
Education	No formal education	26	0.7
	Primary education	459	11.6
	12 years of school – Upper secondary education	875	22.1
	Higher vocational education (vocational diploma)	612	15.4
	Higher education ≤ 3 years (first cycle – bachelor)	769	19.4
	Higher education > 3 years (second cycle – master)	1070	29.0
	Research (third cycle) of higher education	153	3.9
Healthcare education	Yes	1020	25.7
Employment	Full time	88	2.2
	Part-time	113	2.9
	Retired	3688	93
	None of the above	73	1.8
	Not able to work	2	0.05
Self-reported health status	Good	1243	31.4
	Fair	1773	44.7
	Bad	867	21.9
	Missing	81	2
Have received care during the last 2 years^a^	For mental health condition(s)	195	4.9
	For cancer	938	23.7
	For other health problem(s)	3564	89.9
	None	106	2.7
Encouraged to read (by a person)	Yes	378	9.5

^a^ A multiselect multiple choice question.

The data were analysed by one author (IH) in JASP 0.17.1 using descriptive statistics, Mann-Whitney *U* tests and analysis of variation with Tukey post hoc tests. Statistical significance was preset at *p *< .001. No outliers or other data were excluded from analysis. As per cross-sectional research design and non-causal method of analysis, we interpret differences (including their magnitude and significance) between age groups as an indication of change in the perceived affordances of the PAEHR.

## Results

According to the analysis, the most popular affordances of the PAEHR among the respondents (Table 1) were the orientational affordances of its capacity to help improving understanding of personal health issues (mean = 4.397) and get an overview of personal medical history and/or treatment (mean = 4.277) and the cognitive affordance of helping to clarify what the physician had said (mean = 4.119). The least popular ones were related to concerns about inaccuracies (mean = 1.879; cognitive affordance), to share documents with relatives (mean = 1.882; goal-oriented/functional affordance) and because the respondents were unsure if they had received the correct care (mean = 1.933; cognitive affordance).

The respondents also agreed to a high degree that having PAEHR possesses the cognitive affordances of supporting better communication with healthcare professionals (N = 3964, mean = 4.159, SD = 1.128) and helping them trust their healthcare provider more (N = 3964, mean = 4.085, SD = 1.164).

**Figure 2.**
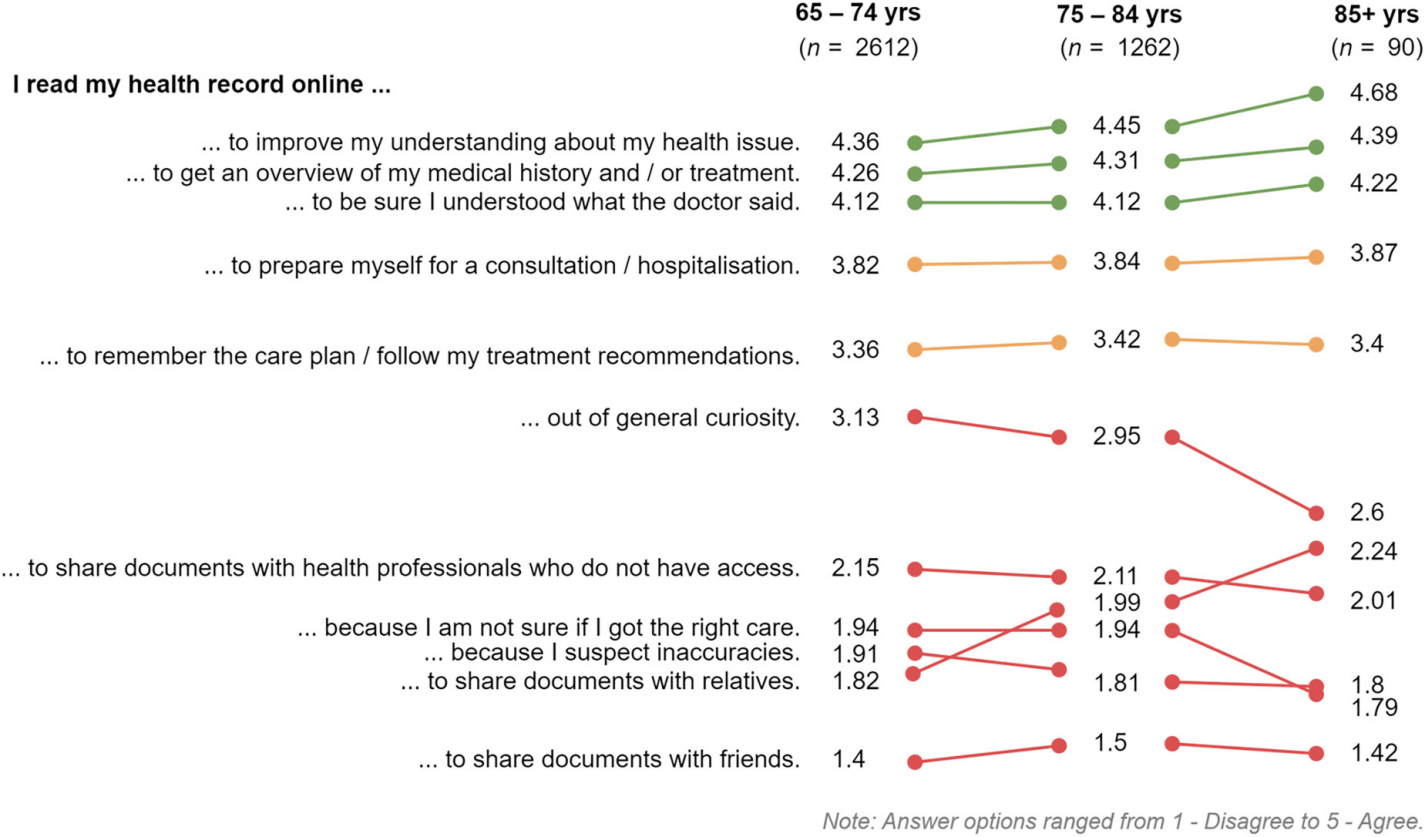
Change in Mean agreement ratings across age groups.

In Figure 2, the mean ratings for using the PAEHR for the three applicable age groups (65–74 years, 75–84 years and 85 + years) are compared. The general tendency is that the most popular, predominantly instrumental orientational and goal-oriented/functional affordances of the PAEHR got higher ratings with age and that less popular reasons decreased in popularity with age. Notable exceptions are the goal-oriented/functional affordance “…to share documents with relatives” where average rating increases with age (1.82 → 1.99 → 2.24) and orientational affordance of reading for general curiosity that decreases with age (3.13 → 2.95 → 2.6).

The association between affordances and having been recommended to read is reported in Table 3. Those who had been recommended agreed to a significantly higher degree that they perceived the affordances of reading their PAEHR to prepare for visits (goal-oriented/functional affordance), remember the care plan or follow treatment recommendations (mnemonic affordance) and to share documents with relatives, friends and health professionals (goal-oriented/functional affordance). Those who had been recommended to read agreed also to a higher degree that PAEHR comes with the cognitive affordances of helping to trust healthcare providers more (*W* = 605,014.0, *p* < .001) and supporting better communication with healthcare professionals (*W* = 606,983.0, *p* < .001).

**Table 3.** Associations between having been recommended to read the online health record and reasons to read it.

Type of Affordance	I read my HR online …	*W*	*p*
Orientational affordance	… to improve my understanding about my health issue.	645,982.000	NS.072
Orientational affordance	… to get an overview of my medical history and/or treatment.	644,389.000	NS.074
Cognitive affordance	… to be sure I understood what the doctor said.	639,675.000	NS.046
Goal-oriented/functional affordance	… to prepare myself for a consultation or hospitalization.	589,034.500	< .001
Mnemonic affordance	… to remember the care plan/follow my treatment recommendations.'	573,574.000	< .001
Orientational affordance	… out of general curiosity.	699,290.500	NS.296
Goal-oriented/functional affordance	… to share documents with health professionals who do not have access.	606,787.000	< .001
Cognitive affordance	… because I am not sure if I got the right care.	664,236.500	NS.481
Goal-oriented/functional affordance	… to share documents with relatives.	561,456.500	< .001
Cognitive affordance	… because I suspect inaccuracies.	673,160.500	NS.809
Goal-oriented/functional affordance	… to share documents with friends.	606,765.000	< .001

*W *: * *Mann-Whitney *U* test statistic; HR: Health record.

The association of respondents’ healthcare education for reasons to read is reported in Table 4. Respondents who reported no formal education in a healthcare profession were more likely to appreciate the orientational affordance of reading for curiosity and the goal-oriented/functional affordance to be able to share the record with friends. By contrast, those with formal healthcare education were more likely to appreciate the cognitive affordance to read because they suspected inaccuracies and the goal-oriented/functional affordance to share the record with relatives.

**Table 4.** Associations between formal education as a health professional and reasons to read the online health record.

Type of Affordance	I read my HR online ..	*W*	*p*	Comment
Orientational affordance	… to improve my understanding about my health issue.	1.544 × 10 ^+ 6^	NS.103	
Orientational affordance	… to get an overview of my medical history and/or treatment.	1.510 × 10 ^+ 6^	NS.746	
Cognitive affordance	… to be sure I understood what the doctor said.	1.536 × 10 ^+ 6^	NS.218	
Goal-oriented/functional affordance	… to prepare myself for a consultation or hospitalization.	1.515 × 10 ^+ 6^	NS.642	
Mnemonic affordance	… to remember the care plan/follow my treatment recommendations.'	1.465 × 10 ^+ 6^	NS.238	
Orientational affordance	… out of general curiosity.	1.636 × 10 ^+ 6^	< .001	No higher
Goal-oriented/functional affordance	… to share documents with health professionals who do not have access.	1.544 × 10 ^+ 6^	NS.138	
Cognitive affordance	… because I am not sure if I got the right care.	1.482 × 10 ^+ 6^	NS.488	
Goal-oriented/functional affordance	… to share documents with relatives.	1.623 × 10 ^+ 6^	< .001	Yes higher
Cognitive affordance	… because I suspect inaccuracies.	1.378 × 10 ^+ 6^	< .001	Yes higher
Goal-oriented/functional affordance	… to share documents with friends.	1.581 × 10 ^+ 6^	< .001	No higher

HR: Health record.

Table 5 reports the associations of the respondents’ self-reported gender and the level of agreement with the reasons to read their health record. Women reported a higher level of agreement with the statements relating to the cognitive affordance of being able to read to be sure they had understood what the doctor said and the goal-oriented/functional affordance of reading because they suspected inaccuracies. In contrast, men agreed to a higher degree that PAEHR affords reading for sharing with both relatives and friends.

**Table 5.** Associations between the gender of the respondents.

Type of Affordance	I read my HR online ..	*W*	*p*	Comment
Orientational affordance	… to improve my understanding about my health issue.	1.953 × 10 ^+ 6^	NS.735	
Orientational affordance	… to get an overview of my medical history and/or treatment.	1.936 × 10 ^+ 6^	NS.401	
Cognitive affordance	… to be sure I understood what the doctor said.	1.797 × 10 ^+ 6^	< .001	Women higher
Goal-oriented/functional affordance	… to prepare myself for a consultation or hospitalization.	1.885 × 10 ^+ 6^	NS.021	
Mnemonic affordance	… to remember the care plan/follow my treatment recommendations.'	1.857 × 10 ^+ 6^	NS.003	
Orientational affordance	… out of general curiosity.	1.996 × 10 ^+ 6^	NS.350	
Goal-oriented/functional affordance	… to share documents with health professionals who do not have access.	2.012 × 10 ^+ 6^	NS.133	
Cognitive affordance	… because I am not sure if I got the right care.	1.864 × 10 ^+ 6^	NS.002	
Goal-oriented/functional affordance	… to share documents with relatives.	2.192 × 10 ^+ 6^	< .001	Men higher
Cognitive affordance	… because I suspect inaccuracies.	1.841 × 10 ^+ 6^	< .001	Women higher
Goal-oriented/functional affordance	… to share documents with friends.	2.121 × 10 ^+ 6^	< .001	Men higher

*W = *Mann-Whitney *U* test statistic; HR: Health record.

The only significant difference between the age groups was that 65–74 years old were agreeing to a higher degree that they were reading for curiosity (orientational affordance) than older respondents (*F*(2,3961) = 8.923, *p* < .001).

Self-reported overall health of respondents was associated with multiple types of affordances. Those with self-reported “bad” health agreed to a lesser degree that they appreciated the orientational affordances of the possibility to satisfy their curiosity (*F*(2,3880) = 11.203, *p* < .001) and to be sure that they understood what the doctor said (*F*(2,3880) = 14.751, *p* < .001), and the cognitive affordance of following up a visit (*F*(2,3880) = 16.828, *p* < .001) than those with “fair” or “good” health. Those with bad health were oriented toward cognitive affordances in how agreed to a higher degree that they used PAEHR because they suspected errors (*F*(2,3880) = 52.393, *p* < .001) and because they were not sure if they had received the right care (*F*(2,3880) = 52.901, *p* < .001) than those with good or fair health.

Those with bad health perceived also cognitive/functional affordances in that they agreed to a higher degree that for them PAEHR afforded to share information with healthcare professionals without access to the record (*F*(2,3880) = 8.285, *p* < .001) and preparing for a healthcare visit (*F*(2,3880) = 11.470, *p* < .001) than those with good health.

Those with good health agreed to a lesser degree that they utilised the orientational affordance of being able to read PAEHR to improve their understanding of their health issues than those with fair or bad health (*F*(2,3880) = 18.316, *p* < .001).

Those with over 3 years of university education agreed to a higher degree that they used the PAEHR to be sure to understand what the doctor said (*F*(6,3957) = 12.194, *p* < .001; a cognitive affordance), to remember the care plan or follow their treatment recommendations (*F*(6,3957) = 15.390, *p* < .001; a mnemonic affordance) than those with higher vocational education, primary school or upper secondary education.

## Discussion

### Affordances of the PAEHR for Swedish older adults (RQ1)

#### Popularity of individual affordances

The PAEHR offered multiple types of affordances among older Swedish adults. The most significant reason to find PAEHRs useful was the capacity to improve patients’ understanding of their health issues, to have an overview of their medical history and/or treatment and to check what the physician had written about their health status. These findings align with other studies conducted in Sweden.^12,37,46,47^ For example, Huvila et al.^64^ found that the main reason for older adults to check their health record was to get an overview of their health condition, to check and verify the details. The findings are also consistent with DesRoches et al.^46^ which found that older patients (in USA) with more chronic conditions were likely to report benefits such as remembering their care plan and checking understanding. Other important reasons to read were to be prepared for consultations or hospitalisation and follow the instructions. Rexhepi et al.^67^ and Simola et al.^68^ similarly highlighted PAEHR use as a memory aid and preparation tool for upcoming visits.

Satisfying curiosity emerged as another major cognitive affordance for reading health records. This correlates with previous findings.^38,47,69^ Sharing medical documents with other healthcare professionals to ensure that right treatment was received was found important also in an earlier Swedish survey.^47^

The least popular affordances were to be able to use PAEHR for coping with suspicion of inaccuracies (cognitive affordance), and in contrast to earlier studies,^70^ the goal-oriented/functional affordance of being able to share health documents with relatives.

Notably, respondents agreed to a high degree that having access to health records supports better communication with healthcare professionals and improves trust in healthcare providers (cognitive affordances). Studies in other countries report similar results; for example, individuals accessing their medical records are more knowledgeable of their health concerns and better able to communicate effectively with healthcare professionals to improve their condition.^52^^,^^71^

#### Contextual and demographic underpinnings of affordances

The role of recommendations from health professionals, fellow patients, family members or friends was associated with Swedish older adults’ tendency to appreciate how PAEHR afforded them to prepare ahead of visits and to plan/follow treatment recommendations. Notably, these recommendations were associated with an inclination to see PAEHR helpful in communicating with their health professionals, similar to studies by Logue and Effken^72^ and Sakaguchi-Tang et al.^73^ Recommendations were also associated with older adults’ propensity to appreciate the affordance of PAEHR to share their medical documents with relatives, friends and health professionals, to help them to trust healthcare providers and that it improves communication with healthcare professionals.

Respondents without a medical educational background tended to access their EHR out of curiosity and shared their records with friends, which may point to the importance of social capital,^74^ that is, relying on networks of people to help understand health information. Conversely, those with medical-related education were more inclined to review their records for potential inaccuracies and to share them with relatives.

Regarding gender differences, women often read their records to ensure they understood correctly what the doctor said and because of suspected inaccuracies. In contrast, male respondents mostly read their notes to share information with relatives and friends. The reasons for these differences are not well understood. The findings from social psychology suggest that females are more cautious than males,^75^ which may help to explain why a higher proportion of female respondents reported checking for inaccuracies. Furthermore, females use health services more than males^76^ and may have greater insight or lived experiences of how breakdowns in communication might arise during clinical visits. Another possible explaining factor, however, with varying evidence documented in the literature,^77,78^ can be that female respondents are taking responsibility for their and their spouses’ and relatives’ to a greater extent than the male respondents.

In contrast to our initial expectations, the age of the respondents had only a minor effect on their motivations to read medical records. The only significant difference was related to the higher role of curiosity in reading notes among older adults in the age range of 65–74 years compared to other age categories.

### Affordance types and older adults’ PAEHR use (RQ2)

While the results confirm earlier observations that PAEHRs provide opportunities, or from the perspective of Affordance Theory, afford a diversity of activities, our study also points to how the affordances-enacted-in-practice differ between individual respondents. In addition to individual variation in the perceived importance of specific affordances, the positive impact of recommending PAEHR use to its recognised usefulness suggests that the perceived affordances are not uniform for all patients. From the Affordance Theory perspective, recommendations appear to increase and broaden PAEHRs’ affordance potency especially toward appreciating their goal-oriented affordances.

Our findings suggest further differences in older adults’ views on the extent PAEHR has goal-oriented or functional affordances^56^ to support specific health and care-related goals, and orientational affordances offering means to satisfy general curiosity and to provide orientation. The goal-oriented affordances are rated higher by those with specific goals and capable of attaining them with PAEHR. In the analysed data, such individuals are marked by their poor health and to a degree, a higher education. Male respondents were also more likely to appreciate goal-oriented affordances, whereas female respondents were leaning toward cognitive affordances. In contrast, the youngest among the surveyed older adults, presumably with less direct healthcare and health information needs, were driven to a greater extent by curiosity. The same applies to those with lower education and lack of healthcare education, presumably less capable of using the PAEHR for specific, practical health goals. In contrast, those with poor health read their PAEHR for functional purposes such as following up a visit and ensuring they understood what the doctor said. Comparably, those with higher education used PAEHR for understanding and remembering details of care visits and care actions more than those with lower education.

On the basis of these findings, we speculate that the orientational, and specifically curiosity-related, affordance potency of PAEHRs is lower than their goal-oriented affordance potency. Also, because there is a reason to believe that curiosity is secondary to specific goals as a propeller of PAEHR use, the affordance potency of PAEHRs is greater for well-educated and sick individuals. However, the possible shift from curiosity-oriented to goal-oriented interest in PAEHRs, supported by general findings on the connections between curiosity and engagement,^79^ might also suggest that satisfying PAEHR users’ initial curiosity could attract people to use it. Therefore, even if it is unquestionably important that a PAEHR is designed with affordances for its users to attain their functional health goals, it is also important that it invites individuals to behaviours that satisfy their broader interests^80^ and “provides or furnishes”^15^ its users curiosity wise.

The differences in how individuals use PAEHRs to remember and understand, align with the notions of cognitive^59^ and mnemonic affordances.^60^ Cognitive affordances of PAEHR can help patients in poor health to think about and understand the professional advice they received. In contrast, as frequent healthcare users, individuals with bad health might not need help in remembering details relating to their condition and care visits. Also, the higher educated respondents valued cognitive affordances, more than their lower educated peers, to help understand what was said during a visit, which can be understood in the light of findings that higher education is associated with better health,^81^ and that those with higher education are also active health information seekers.^82^ However, for more sporadic healthcare users in the group, PAEHRs and their mnemonic affordances can help them to remember care plans and treatment recommendations.

A parallel form of social cognitive, or sociocognitive, affordance that helps patients understand the record is the possibility to share it for additional advice with healthcare professionals. Here, the initial functional affordance as an information source evolves to another, that of a sharing device. Both men and those without healthcare education are more inclined to share PAEHR contents with friends (and for men, with relatives), highlighting the affordance potency of PAEHR to support health management as a distributed, social undertaking where sharing information might be equally important to accessing it.

Considering the theoretical implications of our findings, our study aligns with earlier observations of the Affordance Theory as a valuable tool in understanding technology use and preference trajectories. The results highlight how users find technologies useful in complementary and parallel ways. We suggest in addition that the affordance types can be helpful in developing the PAEHR to afford broader categories of action including specific health and care-related goals (goal-oriented or functional affordances), satisfying general curiosity and providing orientation (orientational affordances), helping to think and understand (cognitive affordances) and to remember (mnemonic affordances) rather than treating all use from a goal-oriented perspective.

While the analysis does not point to radical differences between age groups, the findings, for instance, the decreasing significance of the affordance to satisfy curiosity and the effect of recommendations to use PAEHR, underline that affordances should not be treated in isolation. Instead, we posit that affordances should be linked to each other through trajectories of affordances,^83^ meaning that engaging in technology use can lead to perceiving and actualisation of new affordances. A key is to identify and describe trajectories in technology use and understand how specific affordances lead to others, whether they are depending on age or recommendations as in our study. To design technologies, including PAEHRs, for maximal uptake, it is crucial to identify stepping-stone affordances that are perceivable to potential users and that eventually can actualise affordance potencies and change or broaden them to further affordances. In our study, the curiosity-oriented orientational opportunities for younger respondents and those who had not been recommended to use PAEHR are examples of potential stepping-stone affordances that in time or a specific nudge led to actualising goal-oriented affordance potencies. Similarly, the differences between respondents’ health conditions suggest a potential trajectory from orientational and understanding-related cognitive affordances to goal-oriented affordances. In this trajectory, PAEHR turns to a sharing device, means of preparing for visits and a thinking aid to assess whether the received care or records contents were correct. Finally, a medical-related education is shown as a trajectory from orientational and social affordances of curiosity and sharing with friends to cognitive and goal-oriented ones of finding inaccuracies and sharing with relatives.

Considering these findings, we suggest that designing PAEHR and other eHealth services with orientational affordances can be crucial for the initial uptake. Later on, specific reasons such as higher age and declining health and perhaps corresponding nudges such as education – possibly even less comprehensive training than full medical education – can prompt users to actualise goal-oriented affordances. The same factors appear to be linked to a shift from understanding to thinking-related cognitive affordance potencies with a potential to change, augment and elaborate eHealth use. We posit that identifying additional key prompts comparable to higher age, declining health and education that can actualise affordance potencies along trajectories leading to augmented and enhanced eHealth use is a crucial task for future research and a design task for successful eHealth development.

### Limitations

A major strength of this study is that it is hypothesis generating, facilitating greater understanding about the affordances offered by PAEHRs among older patients. However, it has several limitations. First, the convenience sample limits the generalisability of findings. Although the sample was evenly split between female and male respondents, most respondents reported at least tertiary-level education. This suggests a high degree of formal education, which is associated with higher health literacy. Second, the effect of response bias is unknown, for example, whether those with lower or higher interest in technology and the PAEHR were more likely to respond. Third, due to the explorative nature of the study, scales used to measure affordances were not previously validated. Fourth, the survey was available to *Journalen* users, leaving out those without access to online records. Relatedly, we did not examine income level or chronic disease burden, which may have influenced results. Finally, because the survey was administered in Swedish, immigrants or those lacking fluency may have been precluded from participating.

### Next steps

Further research is needed to understand the functionality of PAEHRs and their interface features associated with ease of use among different patients. For example, those with chronic conditions may be more likely to access lists of prescriptions but, dependent on health literacy, may find them confusing. The popularity of using PAEHR to check for errors among female respondents leads to further questions about the design of error reporting functionalities, and general level of satisfaction among all patients on how and the extent to which mistakes can and should be rectified. The present findings also point to the relevance of identifying new stepping-stone affordances for technologies in different contexts and investigating how they map into specific types of affordances. This could contribute both to the advancement of Affordance Theory and the design and optimisation of technologies for their users and particular anticipated benefits.

## Conclusions

This study shows that PAEHRs have different affordances for various individuals. A PAEHR can have goal oriented, orientational, understanding and thinking-oriented cognitive and mnemonic affordances. The findings indicate further that recommending PAEHR use, medical-related education, decreasing health and to a limited degree ‘age’, influences the affordance potency of PAEHRs.

This study shows how Affordance Theory provides a useful lens to explicate links between individuals’ views of useful functions of information technologies and their different functional opportunities. We emphasise the analysis from the perspective of the affordance that systems offer rather than simply as a matter of personal preferences and perceptions. We propose the new concept of stepping-stone affordances to explicate and understand the role of individual affordances in affordance trajectories and the diverse affordance potencies of technologies, their functions and the available information. Attentiveness to stepping-stone affordances, affordance trajectories and affordance potency of technologies provide guidance for systems designers. This new approach directs attention to system functions that make them useful for their users and why and how the perceived usefulness of functions and available information changes overtime and switches focus from orientation to specific goals and vice versa. A practical suggestion is to engage in identifying prompts or factors that nudge desirable affordance potencies to actualise as functioning affordances for individuals, and co-designing technologies with their different user groups to cater for the trajectories of the affordances these tools have the potential to offer.
